# Supplementary material for: Nilotinib: A Tyrosine Kinase Inhibitor Mediates Resistance to Intracellular Mycobacterium Via Regulating Autophagy
Source: Cells. 2019 May 26;8(5):506. doi: 10.3390/cells8050506 (PMC6562972; doi:10.3390/cells8050506)
Supplement: Supplementary file 1 [file cells-08-00506-s001.pdf]

cells

MDPI

Supplementary materials (Original Article)

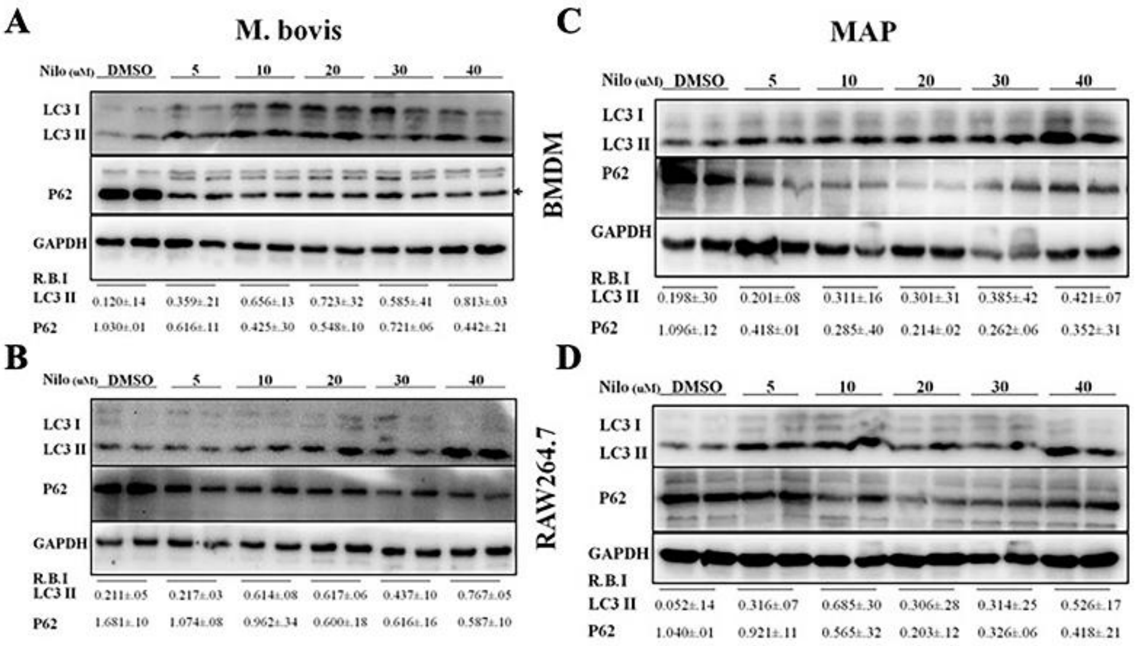

Supplementary Figure1. Nilotinib promotes autophagy markers in *M. bovis* and MAP infected macrophages. (A and C) BMDM and (B and D) RAW264.7 cells were pretreated with DMSO (0.1%) and various concentrations of Nilotinib for 2hours and then infected with *M. bovis* and MAP at the multiplicity of infection 10 (MOI 1:10) for 24 hours. LC3-II and P62 expression was subsequently evaluated by using the western blot (WB) technique. Relative band intensity (R.B.I) values represent the mean ± SD of densitometric analysis of each WB band. Data represent the mean ± SD from three independent experiments.

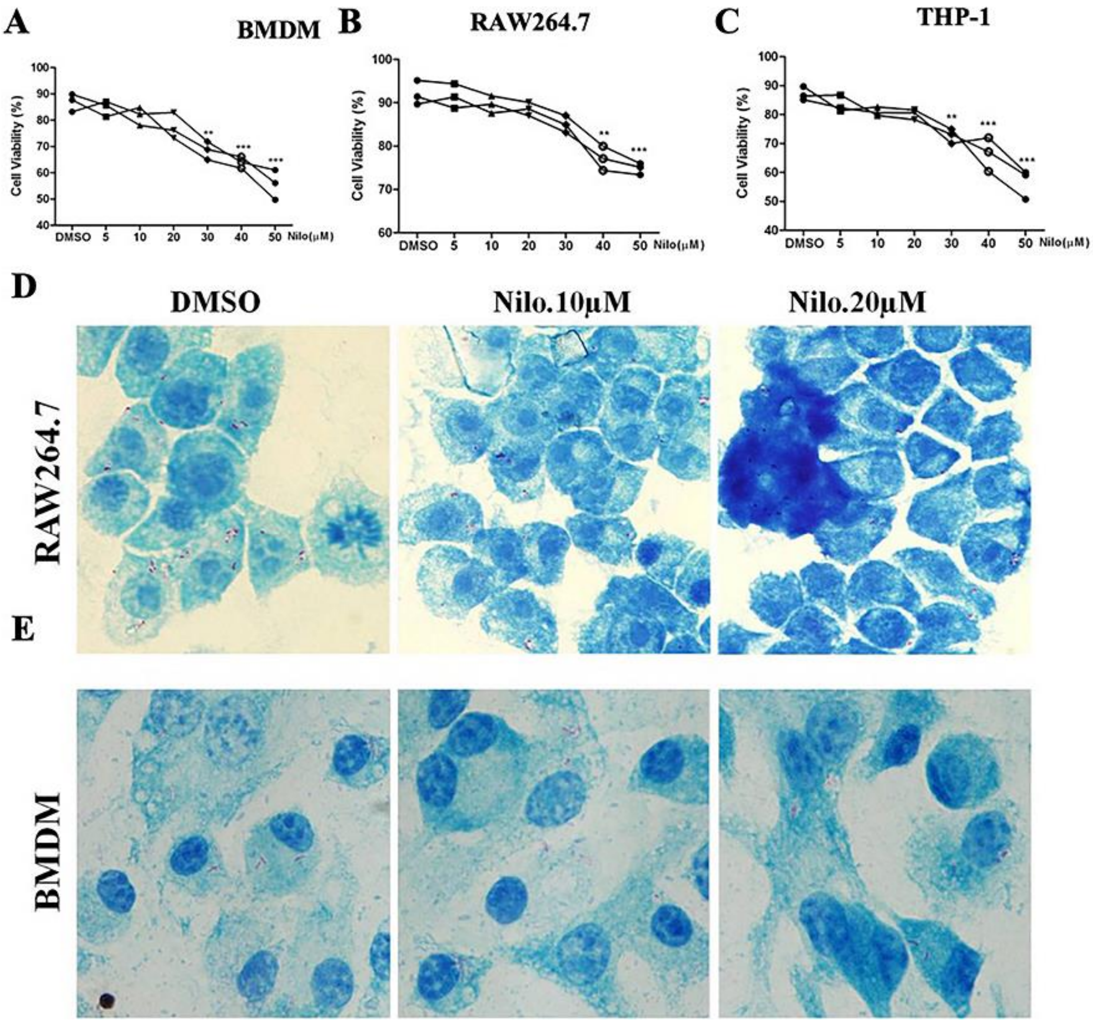

Supplementary Figure 2. The effect of nilotinib on cell viability and phagocytic ability of macrophages infected with *M. bovis*. (A) BMDM (B) RAW264.7 and (C) THP-1 cells were cultured in 96 wells plate for 12 to 18 hours and then treated with different concentration of nilotinib. Cells viability was assessed by measuring Optical density (OD) after addition of MTS reagent with an ELISA plate reader. (D) RAW264.7 and (E) BMDM cells were cultured on small cover glasses in 24-well plates then treated with DMSO (0.1%) or nilotinib 10μM and 20 μM followed by *M. bovis* infection (MOI 1:10). After infection cells were stained for acid fast bacilli and observed under a microscope. Scale bar: 10μm. Data represent the mean ± SD from three independent experiments. **$p < 0.01$, ***$p < 0.001$

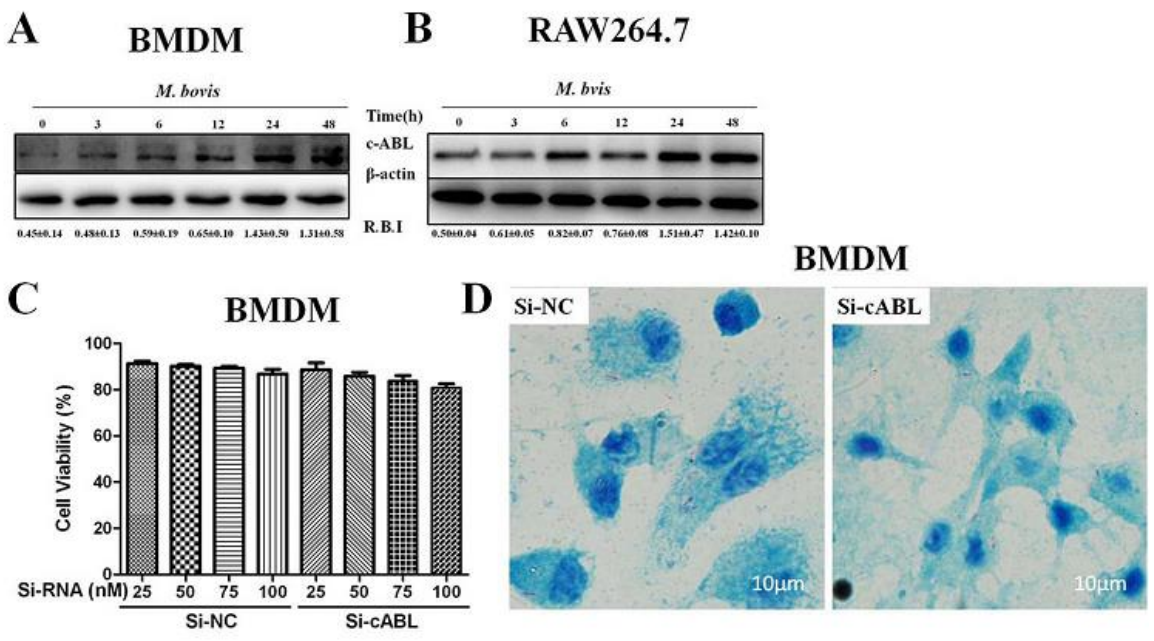

Supplementary Figure 3. The regulation of c-ABL in *M. bovis* infected macrophages. (A) BMDM and (B) RAW264.7 cells were infected with *M. bovis* (MOI 1:10) for indicated time period. Cells lysates were used for cABL protein expression by WB. R.B.I values represent the mean ± SD of densitometric analysis of each WB band. (C) BMDM cells were cultured in 96 wells plate and then transfected with different concentrations of SiRNA control and Si-cABL. Cells viability was assessed by measuring Optical density (OD) after addition of MTS reagent with an ELISA plate reader. (D) BMDM cells were cultured on small cover glasses in 24-well plates, and then transfected with SiRNA control and Si-cABL followed by *M. bovis* infection (MOI 1:10) for 3 hours. Cells were stained for acid fast bacilli and observed under a microscope. Data represent the mean ± SD from three independent experiments.

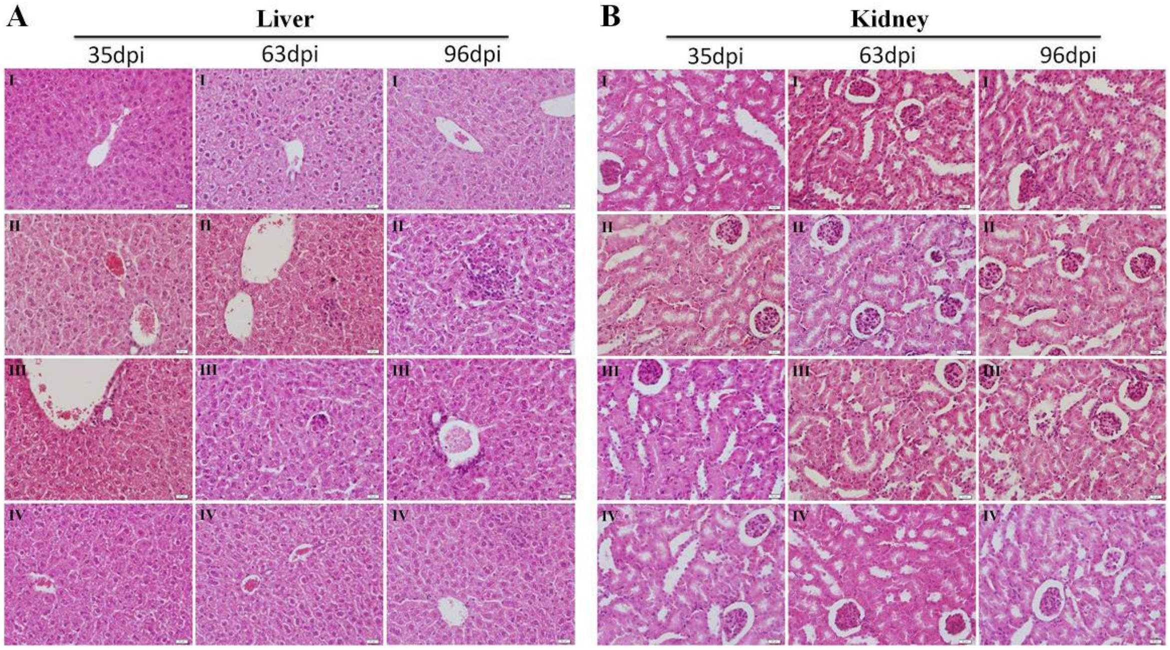

Supplementary Figure 4: The effect of Nilotinib treatment on Liver and Kidney of *M. bovis* infected mice. (A and B) Representative H&E images of liver and kidney histopathology of uninfected mice injected with vehicle and *M. bovis* infected mice injected with vehicle or nilotinib (n = 5). I represent uninfected mice injected with

vehicle, II represent infected mice injected with vehicle, III represent infected mice injected with nilotinib 5 mg/kg, and IV represent infected mice injected with nilotinib 10mg/Kg respectively. Scale bar: 20 μm.

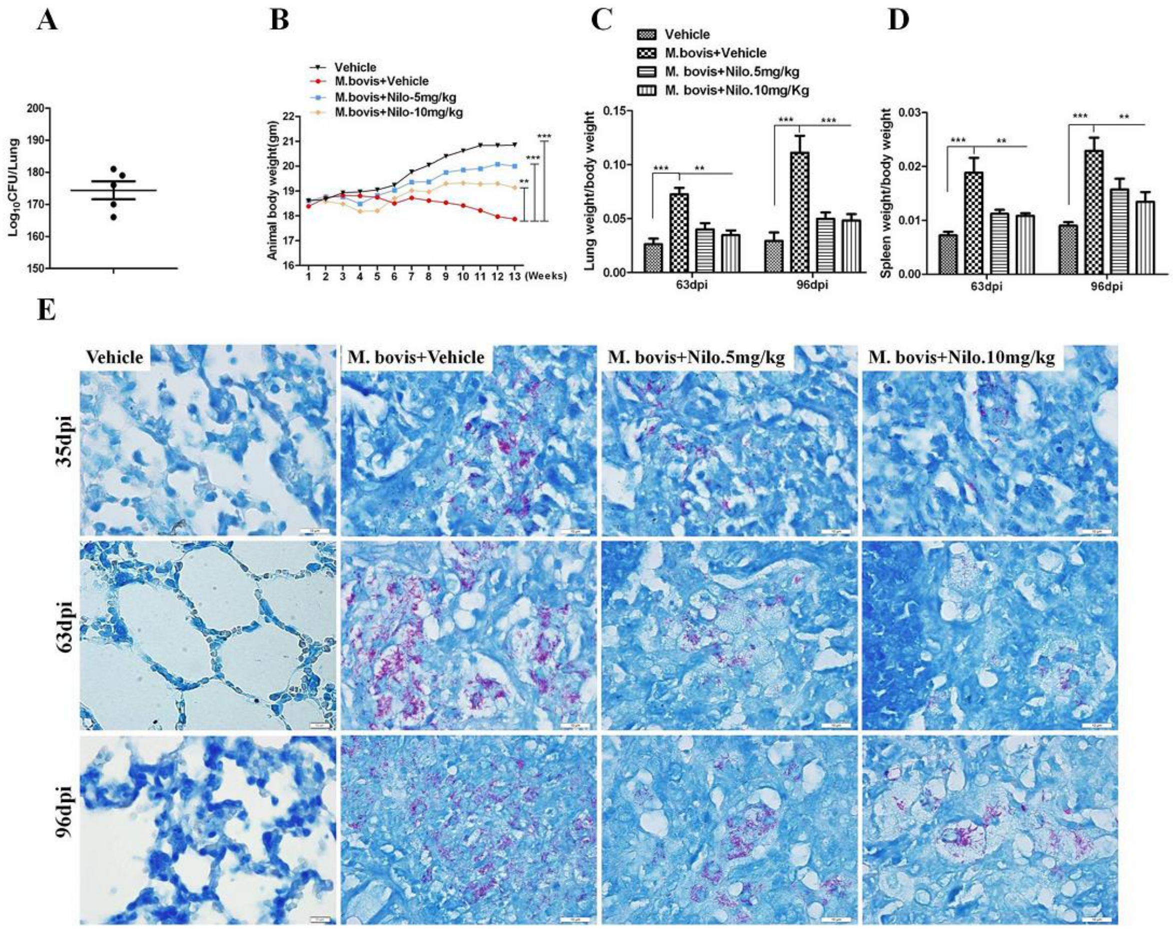

Supplementary Figure 5. The effect of Nilotinib treatment on total body weight and organs weight of mice infected with *M. bovis*. (A) BALB/c mice were infected with *M. bovis* at 200 CFU via intranasal route, after 24 hours of infection 5 mice were randomly selected and slaughter. Lung was harvested; homogenate and tenfold dilution were prepared in PBS. Each dilution was transfer to separate three 7H11 agar plate and CFU was calculated after 2 to 3 weeks of incubation. (B) Total body weight, (C) lungs weight and (D) spleen weight of uninfected mice injected with vehicle and *M. bovis* infected mice injected with vehicle or nilotinib. (E) Representative acid-fast stained images of lung from uninfected mice injected with vehicle and *M. bovis* infected mice injected with vehicle or nilotinib (n = 3). Scale bar: 10 μm. Statistical significance was determined using one-way analysis of variance (ANOVA) correcting for multiple comparisons. $^{**}p < 0.01$, $^{***}p < 0.001$.
